# A qualitative study of home care client and caregiver experiences with a complex cardio-respiratory management model

**DOI:** 10.1186/s12877-021-02251-5

**Published:** 2021-05-07

**Authors:** Connie Schumacher, Darly Dash, Fabrice Mowbray, Lindsay Klea, Andrew Costa

**Affiliations:** 1 School of Nursing, Faculty of Applied Health Sciences, Brock University, St. Catharines, Ontario Canada; 2 Department of Health Research Methods, Evidence and Impact, McMaster University, Hamilton, Ontario Canada; 3 C. Michael G. DeGroote School of Medicine, McMaster University, Kitchener, Ontario Canada

**Keywords:** Home care, Nursing, Self-care, Self-management, Emergency avoidance, Model of care

## Abstract

**Background:**

Home care clients are typically older and have some degree of medical, physical, cognitive or social conditions that require formal or informal support to promote healthy aging in the community. Home care clients contribute a significant proportion of health service use, including emergency department visits. The DIVERT-CARE trial introduced a cardio-respiratory management model to improve client motivation, symptoms and rates of unwarranted health service use. Our objective was to explore the perceptions and experiences of individuals who participated in the DIVERT-CARE self-management support and education intervention.

**Methods:**

A qualitative study was nested within a pragmatic randomized control trial and conducted following a 15-week multi-component cardio-respiratory intervention. A phenomenological descriptive design was employed using thematic analysis. Post-intervention, clients and their caregivers were invited to participate in a semi-structured telephone interview. Interview questions were designed to elicit the experience with the intervention components.

**Results:**

A total of 29 interviews were completed from June 2018 to March 2020 from participants in Ontario, Newfoundland, and British Columbia. Three themes were identified; self-care trajectory and burden of responsibility, learning and behaviour change, and feeling connected pre-emptively to care providers, the information and medical advice, and connection through the therapeutic relationship.

**Conclusions:**

Home care clients experience unique challenges in managing cardio-respiratory related chronic disease. Home-based interventions fostered a therapeutic relationship of connectedness while equipping clients with necessary knowledge and skills. These results inform recommendations for community nursing, and home-based self-management supports for older community-residing individuals.

**Supplementary Information:**

The online version contains supplementary material available at 10.1186/s12877-021-02251-5.

## Introduction

Home care (HC) clients are a large and expanding group of medically complex older adults with poor access to effective chronic disease management [1]. Limited community-based resources and budgetary constraints leave many HC clients with unmet care needs [2]. These individuals frequently turn to the emergency departments (ED) for medical care, contributing twice as many visits when compared to other older adult populations [3]. Time pressures and high patient volumes hinder emergency clinicians from addressing geriatric complexity and chronic disease management in the ED [4, 5]. Greater emphasis on improving community-based disease management and service integration is recommended to support the multifaceted needs of older adults and prevent unwarranted ED visitation [6].

HC clients with cardio-respiratory disease or symptoms (e.g., chest pain, shortness of breath or dyspnea, dizziness or arrhythmias, etc.) are at greater risk for clinical decompensation and frequent health service utilization. Internationally, older ED patients share characteristics of frailty and functional dependency, with one third presenting to the ED with shortness of breath [7]. Prevalent conditions and symptoms on presentation to the ED include heart failure, chest pain, pneumonia and respiratory complaints of shortness of breath [8]. For the older person, ED visits are associated with a higher risk of adverse events, return visits to the ED and fragmented care [9].

To better identify and support these individuals in the community, we are currently conducting a multi-site pragmatic cluster-randomized control trial (RCT), herein referred to as the DIVERT-CARE Trial (NCT03012256). The objective of this trial is to evaluate the effectiveness of a cardio-respiratory disease management model for improving client motivation, reducing symptoms, prolonging time to unplanned ED visits and health care cost [1]. HC caseloads were randomized to either receive the DIVERT-CARE intervention or the standard of care. Clients were assessed using the Resident Assessment Instrument for Home Care (RAI-HC) which identifies those at risk of an ED visit through the Detection of Indicators and Vulnerabilities of Emergency Room Trips (DIVERT) Scale [10]. Enrolment occurred as new and existing clients had a RAI-HC completed. Clients who met the RCT eligibility criteria were enrolled at a rate of approximately 1–2 per week per caseload. In treatment clusters, clients identified as high-risk for ED visitation through the DIVERT scale were offered a package of evidence-based interventions [11]. The intervention components were conceptualized using Wagner’s Chronic Care Model (CCM), a client-centred and integrated health systems approach to improve self-management [12]. There were six client-facing components including a scheduled 15-week nurse-led self-management support, access to a staff helpline, education on vaccines, advance care and goal planning, medication reconciliation with a pharmacist and documented recommendations to support continuity-of-care in the community [1, 11]. The two staff-facing components were inter-professional team case rounds and use of the situation-background-assessment-recommendation (SBAR) technique. All client-facing intervention components were offered and clients could select involvement in one or more of the components. Nurse-led self-management support consisted of four home visits and four telephone assessments over a 15-week period. As part of the intervention, HC clients received congestive heart failure (CHF) or chronic obstructive lung disease (COPD) zones reference sheets [13]. The zones are one-page symptom monitoring decision aids for CHF and COPD.

The integration of qualitative research methods in clinical trials is common, as they shed light on contextual factors surrounding trial findings, methods employed, feasibility and participant experiences [14, 15]. The DIVERT-CARE Trial results will aim to establish the effectiveness of the model in a future publication. However, understanding the participant experience is equally important, as HC clients have a primary role in their care and self-management. Self-management was prominently featured in the intervention in the context of preparing the HC client to manage cardio-respiratory symptoms. Actions taken and learnings during self-management are highly person centric, thereby reinforcing the need to explore the individual’s unique perspectives as they encountered the intervention [16]. These learnings can influence future self-management interventions as they are designed and delivered to best support the participant. Our objective was to explore the perceptions and experience of HC clients with self-management of cardio-respiratory symptoms related to chronic diseases after participation in the DIVERT-CARE intervention.

## Methods

We conducted a phenomenological descriptive study that was nested within the DIVERT-CARE trial and conducted at the end of the 15-week client intervention period. A phenomenological descriptive approach was used to explore and describe meaningful experiences as it is lived and experienced by the person [17, 18]. Self-management is a multi-dimensional concept where the client participates in activities that align with their perception of wellness, as a temporal and subjective experience. Self-management as a phenomenon was conceptualized through the lens of intentionality, where consciousness is directed towards something to create meaning [19]. The research question and interview guide were formulated to elicit the meaning of self-management for those with cardio-respiratory symptoms as experienced with the DIVERT-CARE intervention. We sought to obtain detailed descriptions of a specific experience and reduce to common meanings across participants [20].

The qualitative study was conceived and designed in conjunction with the trial protocol [1]. This study was performed in accordance with the Declaration of Helsinki and approved by the Hamilton Integrated Research Ethics Board, Island Health Research Ethics Board, and the Newfoundland and Labrador Health Research Ethics Board. This study was funded by the Canadian Institutes of Health Research (grant # 148,933).

### Research Question

What are the experiences and perceptions of HC clients or their caregivers concerning self-management through the various components of the DIVERT-CARE cardio-respiratory management model?

#### Participants and Study Setting

The study took place in three publicly funded HC organizations located in three Canadian provinces: Ontario, Newfoundland and Labrador, and British Columbia. HC caseloads are managed by designated health professionals with caseloads typically ranging between 80 and 120 clients. HC clients eligible for the RCT were assessed to be within four high-risk DIVERT scale scores. The common characteristics included a recent ED visit or hospitalization, presence of a cardio-respiratory symptom (chest pain, shortness of breath, or dizziness), and underlying coronary artery disease, heart failure, or COPD. Following a 15-week community-based multi-component cardio-respiratory intervention, HC clients or their caregivers were recruited to participate in a telephone-based interview. A purposive sample strategy was selected to best represent understanding the experience clients and/or their caregivers had with the intervention components. Participants were eligible for study inclusion if they participated in the intervention, were English speaking and provided a contact telephone number. Where the recipient of care was not autonomous in their self-management, their caregiver was approached to gain a contextual understanding of their experience. Caregivers approached were the primary provider of self-management activities and had participated in the 15-week education portion of the intervention. Participants who consented to further contact post-intervention were contacted by the research coordinator by telephone (DD, LK). Enrolment in the RCT was over a time period of approximately 18 months, with some variation between sites due to variation in the study start date, demographics and caseload size. Although there is no ideal sample size, we estimated a sample size of at least five per site based on recommendations of a minimum of five participants for phenomenological studies [21, 22]. For this purposive sample, we aimed for representation of both males and females, and representation from all three sites participating in the DIVERT-CARE intervention. Interviews were completed from June 2018 to March 2020.

#### Data Collection and Analysis

A date and time were arranged to complete the interview with a research coordinator (DD, LK). Research coordinators for this qualitative study did not have a pre-existing relationship with the participants in the RCT. Participants provided informed verbal consent that was audio-recorded prior to the interview. Semi-structured interviews with clients or caregivers were conducted using an interview guide (found in Additional File 1). The interview guide was structured to elicit experiences with the DIVERT-CARE intervention components, changes to managing their health, communication with care providers, and areas for improvement to better suit their care needs. Throughout the data collection period, interviewers kept a reflexive journal to mitigate bias by noting down reflections on what was shared during the interview and what was important to the participant. These notes were brought forth and used during analysis. Interviews ranged from nine to 57 min, averaging 27 min. For one participant, responses were brief resulting in a short interview as the participant did not elaborate upon probing attempts. Data were transcribed verbatim and anonymized for analysis. Participants were not offered reimbursement for participating in the RCT or qualitative study.

Qualitative data were housed in QSR International NVivo 12 Pro. We analyzed the data using a phenomenological philosophical lens and thematic analysis technique, following these steps: familiarization with the data, generate initial codes, search for themes, review themes, define and name themes, and produce the report [23, 24]. To validate coding, each transcript was independently read and analyzed by two members of the research team (CS and LK). The process involved line-by-line coding of the first five interviews, after which the coding was compared, and the codebook was developed. All other interviews were then coded independently, and the codebook was further refined [25]. Frequent meetings were held between two researchers (CS, LK) to discuss the meaning units, alignment with the codebook, and any emerging codes. The codes were examined with a constant comparison approach by four researchers (CS, LK, DD, and FM) to discuss and refine the emerging themes [26]. The fourth researcher (FM) was brought in for the purpose of triangulation and independent analysis. Themes were derived from the data, by focusing on patterns and descriptions of the experiences related to the research question. All data were given equal consideration during analysis, and codes were reduced into themes that were adequately descriptive. Quality of data was the determination for saturation, where themes illustrated contextual properties and were demonstrated across the sample [27]. Thematic saturation was reached during analysis where the research question was answered through these recurring themes.

Reliability and validity were checked by two researchers (CS, DD), where analysis and interpretation were continuously monitored and systematically compared back to the data [28]. Measures taken to ensure reliability included meetings between each interview to discuss the interview guide and probing questions. Coding and new codes were discussed at weekly meetings with a review of codebook definitions and changes to the coding structure. NVivo word queries were run along with cross-case analysis queries looking for patterns and verifying representation of codes across participant responses [29]. NVivo analysis functions served two purposes: to check replication of manual coding and to check synonyms and word associations for missed representations. Representative quotes were selected from the interviews to highlight key findings. We used the Consolidated Criteria for Reporting Qualitative Research (COREQ) checklist to guide the reporting of this study [30] (Additional File 2). Specific strategies used to attain trustworthiness included member checking post interview, peer debriefing, audit trails, and analysis of negative cases [31]. Member checking was completed immediately following the interview as this provided an opportunity for the participant to clarify, elaborate, or rephrase their comments. This helped verify data and minimize misinterpretation. We did not elect to complete member checking after thematic analysis, but instead completed at the end of the interview to prevent loss of contact during hospitalizations or due to death given the HC client population this study included. Peer debriefing occurred at regular intervals while reducing codes into themes as an opportunity to minimize misinterpretation and consensus building on meanings. Memos were used to record coding decisions and provide an audit trail.

## Results

### Participants

A total of 70 individuals consented to be approached for follow-up contact. Of these, 56 were purposively selected based on our criteria of site and sex representation. We contacted 56 individuals to request participation in the present study. We were unable to reach 14 participants and 13 refused to participate, reporting a lack of time or interest as reasons for refusal. Out of those contacted, 51.7 % (*n* = 29) agreed to participate. We had consent from six males and 23 females; 20 participants were HC clients and nine were caregivers. HC clients whose perceptions are reported in this paper were aged 51 years to 98 years with a mean of 78 years. 19 interviews were from Ontario, four from Newfoundland, and six from British Columbia. Three themes emerged from the thematic analysis: self-care trajectory and burden of responsibility, learning and behaviour change, and feeling connected pre-emptively to the care provider, the information and medical advice, and connection through the therapeutic relationship. Representative quotes are included below with pseudonyms for participants and were classified as ‘C’ for HC clients and ‘CG’ for caregivers, and as one for Ontario, two for British Columbia or three for Newfoundland study sites. Supplementquotes are provided in Table 1.

**Table 1:** Themes and Supporting Quotes

Theme	Additional Quotes
Self-care Trajectory and Burden of Responsibility	*Yeah, and like, you know, a lot of people, like a lot of older people, they live on their own and they don’t get the proper help that they should because they don’t either call or they don’t know, you know until it’s too late. So and the kids don’t, a lot of kids don’t care anymore.* John (CG, 1) *And so it makes me kind of frail, you know.* Alicia (C, 3) *I think I’m [pause] I’m not gunna get better. Because, like I say, but I know, I just hope, I just pray that I don’t get worse.* Susan (C,1)
Learning, Changing Behaviour, and Confidence	*Because I actually listen more and try to understand more and take advice more than I did before*. John (CG, 1) *Well, I feel very confident that they know what they’re doing and– I don’t know, I just feel confident. Maybe that’s a result of it. Well, I just feel more aware around when my blood oxygen is low, I don’t breathe very well.* Alisha (C, 2) *One of the things that she helped me out with is she says “do I weigh myself?” I said “well no” [laughs]. She said, “well that’s a very good idea because now that you’re on fluid pills or water pills, that’ll tell you if everything is working or not because you’ll lose weight. If your weight goes up again, it means something is not right or something you’re not doing right and you have to get in and get it checked out”. So started weighing myself in the morning when I get up and weighing myself in the evenin’ before supper*. Bob (C, 1) *Well like I said before, once you know the drugs you’re taking and you can identify with ‘em and you know rough, even if you have no medical background … they make it clear enough to you which pill is for which – what it’s supposed to be doing, then you know which ones for sure you can take or can’t forget to take it.* Bob (C, 1) *Well, every zone tells you why you’re… ya know, obviously if you’re in the red zone it’s not good at all, but at least if you’re in the yellow zone it means to let you know you need to watch something or you need to go back to your doctor or … like there’s a problem coming. Oh, I understood that if you got, if you were silly enough to get back in the yellow zone somewhere you go straight to emerge [/Emergency Department/]*. Bob (C, 1) *If you take the medications and follow the instructions they are things that are liveable. Could be worse [laughs]. These are things that can be taken care of in your life, and you can manage ‘em and you can go on and live your life as long as you follow the rules and take the pills and whatever.* Bob (C, 1) *She was really good. She got me to understand how to do it properly. I was doing it the wrong way*. Mary (C, 1)
Pre-emptive Connection to DIVERT-CARE Professionals	*She dealt with and checked out and made sure that I, you know, all my meds — I kept him in a blister pack, and she helped me; showed me some exercises and basically checked out the type of diet that I was on. And yes, I would say initially she came probably over a course of a few days and then she would check up on me primarily by phone. And of course, she was available if I had any problems.* Jim (C, 1) *Just checking up on him was good*. Carol (CG, 1) *She’d be by the next week at a certain date at a certain time and she’d always be there on time. She checked the blood pressure and asked me general questions and how I figure I was doing.* Bob (C, 1) *They’re going to check in and that also, that was, uh, connecting down to the office. It was hooked up so it would go down to their computer. [home monitoring] Blood pressure run up, they’d phone, or down or if something might be wrong on the readings, they’d phone.* Maureen (C, 2)
Connected to Information and Medical Advice	*It made it a little more understanding and, you know, things that are happening and that.* Karen (C, 1) *Well, she explained everything. Like, you know, we wrote everything all down. She explained about watching the weight. She showed me all the danger zones and what I should watch for.* Tammy (CG, 1) *She could tell if we were able to work on our own or if we need that extra assistance because of not being savvy. But you know she did everything: she simplified things, making sure that we were absorbing the information.* Donna (CG, 1) *I like to be independent to a certain degree and she allows me that. Doesn’t try to tell me “Don’t do that because you’re not supposed to”. It’s a form of “let me try something”. If I can’t do it, I know she’s there to back me, to help me.* Sarah (C, 1)
Connection through Therapeutic Relationships	*And [Nurse] was very patient, very understanding that way. She was a tremendous asset. I have to say, she was really, really awesome*… Karla (CG, 1) *For one thing, it’s wonderful to talk to somebody else. Um, they’d come in and it’s like they’re friends, you know?* Alisha (C, 2)

### Self-care Trajectory and Burden of Responsibility

The interview guide begins with questions about the participant’s conditions and their role in their care. The intervention is comprised of multiple components that aim to enhance self-management and having the person describe their condition and caring for their condition facilitated crystalizing what may have changed. Participants described their experience with the DIVERT-CARE intervention within the context of self-care and their journey managing chronic disease symptoms and treatment. We asked how managing their care had changed following the provision of health education and support in their home. Many described this change by first elaborating on their experiences caring for themselves over the course of their disease, including perceived capabilities, feelings, and uncertainties. Several HC clients and caregivers acknowledged a lack of confidence in their ability to provide self-care.

‘*Well see, another bad mistake with a lot of people, and I was like, feelin’ guilty of that myself, you think you know all of it. But never be afraid to find out more because, ya know, there’s times over the years, not in the last couple of years, but over the years since 1996, there’s things that I’ve been doing that I’m not doing it right. Like I thought I was doing it right*’ Bob (C, 1).

The daily toll and responsibility of caring for one-self and navigating changes in health were likened to feelings of burden. In addition to feeling burdened Bob describes feelings of guilt for not performing self-management correctly in the past. Common daily symptoms that were reported included shortness of breath, and edema, most often seen in the lower extremities. Some participants highlighted that shortness of breath seemed difficult to manage and lacked distinct features where actions should or could be taken. For instance, one respondent described that their bucket was full, giving a sense that they were overwhelmed with ongoing monitoring and self-care responsibilities. An inability to alleviate symptoms was common among participants and evoked feelings of fear, uncertainty, and inaction.

*‘I have shortness of breath again and I have my feet are swollen. I never thought nothing of it because of the fact that I’ve always had this shortness of breath, right?…So, anyway, I’m diagnosed with that now, so I don’t know what’s- what’s going to be next. My bucket is almost full.’* Keith (C, 3).

‘*It scares the hell out of me sometimes. Yeah, I get really scared and then I just, I think – what am I going to do, like what am I going to do? And – I’m not going to treat this anymore because it is too much, and uh, like, I can’t breathe [crying]…Not everything, but maybe one of the most important things, you forget. ‘Oh God’, you know? What am I going to do? What should I do? I forget, I can’t remember.*’ Lori (C, 1).

Many reported concerns about the impact that their care was having on the lives of their families, and that they desired to take sole responsibility for their care. To illustrate, Jim (C, 1) shared that his ‘*kids are married, they have kids and grandkids and you know. I don’t want to become a burden, as far as their lives are concerned*.’ There seemed to be a hesitancy to rely on others, and a clear desire for more information. Participants freely discussed the work or burden of self-management, as it affected themselves and the persons in their lives.

### Learning, Changing Behaviour, and Confidence

The second theme arose from descriptions and perceptions of self-care during the course of the DIVERT-CARE intervention. Participants were asked a series of questions for each of the client-facing components of the intervention, prompted by an examination of the influence that the intervention component had on daily care management. Most described they learned something new about their medical condition, medications, and managing symptoms. Several elaborated further on the strategies they used to monitor their condition, their increased interest in their own care, and using the zones decision aids. Decision aids were incorporated into the 15-week self-management component, where clients were taught how to recognize early symptom warning signs.

Karla (CG, 1) articulated her increase in awareness over her condition: ‘*I would have to say it would open up my eyes an awful lot of different things … it made me more… attentive and… being more aware and to watch for certain things… which was a real good thing because I had no idea, ya know*?’ Similarly, Joanne (CG, 3) noticed a change in Keith’s (C, 3) attitude towards his health: ‘*it seems like if you’re talking about anything, uh, about his health or anything, like he’s right in there, he wants to talk about it, whereas before he was kind of like he would shy away from that conversation type of thing*.’

Self-management education included knowledge of medications, where a pharmacist completed a comprehensive medication review along with education, ensuring that the client was taking medications correctly as well as making dispensing recommendations. Some respondents described knowledge that they had learned, and were able to teach-back the content as noted by one participant describing inhaler utilization:

*‘I’m pretty well set up. I was to use it twice a day. To keep the little, um, alveoli or whatever call it open; the little sacks in your lung. Keep them open and I don’t use it as often now because I don’t really… It does help if I have a lot of mucus.’* Elizabeth (C, 2).

For some, the information they received was new, and the majority reported gaining knowledge on how their medications worked and strategies to manage symptoms of shortness of breath and edema. However, there was one exception where a HC client had previously participated in education for COPD. He articulated new knowledge regarding the transient nature of chronic diseases and a new awareness of functional impairment.

*‘Obviously with emphysema and COPD it’s not likely to improve, but it’s very nice if you can get it stabilized so it doesn’t deteriorate. That’s right. I was very well informed. You know, right now I’m down and… you know, it fluctuates a bit around 35 % lung capacity which, you know, as long as they don’t over exert myself in high humidity or even at any other… I notice I have limitations, but I know what they are, and I know what to do when I’ve reached them so.’* Mark (C, 1).

Mark also brought up a unique perspective, as he already felt aware of some aspects of the intervention due to previous participation in another self-management program. Despite previous exposure, Mark identified it was still useful to participate and re-fresh on self-management principles. For most of the other participants, the program was their first exposure to self-management principles.

### Feeling Connected

 As each of the intervention components were discussed, we found an important theme woven throughout the participant’s interviews describing their experience, which was a feeling of connection. These findings are presented with three sub-themes: pre-emptive connection to the care provider, connected to information and medical advice, and connection through the therapeutic relationship.

#### Pre-emptive Connection to DIVERT-CARE Professionals

HC clients and caregivers in the study reported a feeling of security and connection, which was expressed as continuity and initiation on the part of the care provider. A recurring statement across interviews was appreciation for consistent follow-up regarding health status, whether via an in-home or phone visit.

*‘It was nice to have her come in and… just to check the heart and lungs, and make sure the medications were okay and so on. We’re just grateful that someone was coming in and that someone was available if we needed them!’* Paul (C, 1).

The importance of follow-up on timing to diagnosis and intervention was best illustrated in the case of Keith, as told by his caregiver:

*‘And-and she-she comes in every couple of weeks or so, check it all out and she was floored that his pulse and everything was so low. But I feel like you don’t know. What if she didn’t come in? It was the perfect time. And God love her. Like if it wasn’t for her coming in and doing that, we wouldn’t have known that like, you know, he’s always had a little bit of issue with his pulse and stuff, but not down in the 30s and that. And with her helping me with that, we tried to figure things out. She got a hold of the doctor about it. They made, um, then the doctor made an appointment with us. He went in, uh, and he ended up getting, uh, a pacemaker put in.’* Joanne (CG, 3).

One exception to this theme occurred when additional services were coming into the home. Maureen (P, 2) expressed that a high frequency of visits led her to book visit-free periods so she could have ‘*a couple of days to [her]self*’. The multitude of visits in this case was perceived as a *‘nuisance’* and not productive.

#### Connected to Information and Medical Advice

Over the course of the interviews, many referred to the provided resources, such as the zones decision aid and client education booklets. Participants described scenarios where their health care provider reviewed information and contextualized it to their reality.

*‘I got heart failure zones and I’ve got uh, private community services. I do have that big book that we all got, that health thing that tells you what to do in case of stuff. That big thick one. Remember we got that, who to call and everything, and I look, I refer to that once in a while.’* Ellen (C, 2).

However, many reported requiring additional support to digest the information in order to use the information independently. When time was spent reviewing the information and verifying understanding, participants had more confidence in decision-making.

*‘But I think it was certainly beneficial to have someone that I could discuss my concerns with, such as just giving me an explanation. I know at the time I found it kind of strange that I was on a diuretic and at the same time I was also on a medication to control the frequency of my bladder. I thought, you know, seems to me like you know these two things are working in opposition*.’ Jim (C, 1).

Two intervention components that worked well together were the 15-week self-management by nursing and pharmacist’s medication review. The provision of a medication reconciliation by pharmacists, and reinforced by nursing staff, was also reported to be beneficial. Lori (C, 1) stated, ‘*I don’t think I would’ve been off as much medication as I am now without all the help I got to do it*.’

#### Connection through Therapeutic Relationships

The final sub-theme is best described as the emotional connection to the DIVERT-CARE health care provider. Regular visits that occurred over an extended period facilitated therapeutic relationships, and in some cases an emotional bond. Participants expressed that they were satisfied with the program and the health care professionals involved in delivering the intervention components. Words commonly used to describe their interactions included ‘*comfort*’, ‘*caring*’, and ‘*understanding*’.

*‘She was good. She was really good, I must say’.* Keith (C, 3).

*‘It was, well I would say a comfort. It was, yeah. It was a [pause]. You know, I think we’re blessed to have the care that we get*.’ Carol (CG, 1).

Participants felt an attachment expressing that they looked forward to the visits and felt comfortable having candid conversations to discuss their cares and concerns. Even participants, like Mark (C, 1), who did not feel like they needed a lot of support expressed that the intervention was mutually beneficial: ‘*There was nothing I didn’t like. It worked alright for me. Anyway, you know, I’m not sorry I went through it, it gave them some insight*.’ The burden of self-care was apparent, and the gratitude shown by the participants demonstrated the utility and benefits drawn from this intervention. As eloquently summarized by John (CG, 1), ‘*She treated mom like a person. Not a number’.*

## Discussion

We provide a current perspective from HC clients and/or their caregivers with cardio-respiratory symptoms in a self-management program. Our study highlighted pre-intervention experiences of uncertainty regarding best practices in existing self-management of chronic diseases, such as COPD, CHF, and comorbid diabetes. Self-management was also described as burdensome and overwhelming prior to participation in the DIVERT-CARE intervention. Our multi-component intervention provided participants with valuable information that was participant-friendly, client-centered, and actionable. As participants reflected on the support they received, a dominant theme of feeling connected illustrated the importance of person-centered approaches. These findings highlight a need for client education and additional support with daily chronic disease management. An assessment of baseline health knowledge is essential in HC clients, as this information can be used to tailor educational content, knowledge translation strategies and learning needs.

Our findings and results are predominantly in reference to the 15-week nurse-led self-management component. Of the six client-facing components, nurse-led self-management had the longest duration and are what participants recalled most. Other components were embedded into the nurse-led self-management where participants may not have differentiated between them. For instance, vaccine offerings were single events in time, while medication review and advance care planning were conducted during one or two visits. The benefits of a medication review and vaccination may have been less memorable or not viewed as discrete components to the overall self-management experience. Similarly, the hotline was not prominently mentioned or used as many expressed not needing to use it and preferring to reach out to their care provider directly. As the CCM suggests, this is not a negative finding as integrated system changes may not be overtly recognized by the client, however, they are still delivered to the client [12].

Following participation in the DIVERT-CARE intervention, there was consensus among participants about the utility of health education and client-decision aids. Participants identified the benefit and need for additional health education in the home setting, as it was reported to increase or reinforce health knowledge. However, participants were reluctant to share their self-management journey and challenges to both loved ones and care providers. With loved ones, many chose to navigate their self-management on their own via trial and error, rather than burdening their loved ones. Additionally, participants struggled with discerning severity of symptoms that required medical attention and hesitated to relay gaps in knowledge and care needs unless prompted by a healthcare provider. Older adults, similar to our sample, have reported difficulty in decision-making during complex medical situations and express concerns about cognitive capabilities [32]. In a systematic review of barriers and facilitators of self-management, it is reported that expert self-managers become experts on their own through trial and error, most often during critical times, where behaviours are adapted to achieve a desirable result [33]. Our findings suggest that pre-determined and consistent support in self-management is a key component that could minimize anxiety about self-care. Additionally, more frequent client engagement increases confidence and is associated with lower health service use [34]. According to Wagner’s CCM, self-management support is the most efficacious intervention for improving health outcomes [35]. Although many self-managers learn on their own, it is important to consider the stress and burden associated with caring for oneself. We recommend establishing a network of support with informal and formal care providers for ongoing self-management navigation.

A particular component of health education found to be beneficial was the use of concise and actionable decision aids (e.g., the zones instrument). This tool empowered participants to confidently gauge the severity of their disease state and to act accordingly, which leads to a decrease in miscommunication between providers and clients [36, 37]. In our study, participants appreciated having 24-hour clinical support available to confer and discuss their understanding of the symptoms they were experiencing and the appropriate clinical interventions moving forward. Based on these findings, we recommend that models of care in the community provide consistent messaging across all educational resources and are congruent with clinician recommendations. We do recognize that HC participants in our study received more than the standard of care, as most services are reactive, and task based. However, there is an opportunity to use existing HC tools, such as the DIVERT scale in the RAI-HC, to support early identification and management of symptoms prior to deterioration and disease progression [10]. Using existing tools within the system can facilitate day-to-application and adoption, while remaining cost-effective [12].

 To increase our understanding and achieve adequacy of data on self-management and experience with the intervention, we made the decision that all intervention participants would be invited for a qualitative interview. Our findings indicated that for the majority of the participants, this was their first exposure to a self-management program. In a negative case analysis, Mark (C,1) had previously had experience with self-management education in another program. Although Mark accepted the DIVERT-CARE intervention, he expressed already being aware of what was covered and that others who have not had self-management would benefit from participation. Even though he did not need to participate, he valued the re-fresher. Previous research has recommended that self-management programs should be tailored to the real needs of the person [38], offered multiple times to facilitate integration of knowledge and behaviour change over the natural course of disease [39], and that regular professional contact and check-up is beneficial [40]. For persons with chronic illness where cardio-respiratory symptoms are involved, self-management programming should not be portrayed as a one-time event. Our intervention was set up to be delivered as a one-time offering, and the negative case analysis and literature suggest that education needs to be tailored to the change in disease progression and should be offered on a continual basis.

An interesting theme that emerged from this work was the perceived intimacy of therapeutic relationships that developed as a result of having consistent health care providers in the home. Therapeutic relationships may have been forged due to the intervention being 15-weeks of visits with one provider. Continuity-of-care was an essential component of the DIVERT-CARE trial as it improves client outcomes and allows for the fostering of therapeutic-client relationships [11]. Participants appreciated the option of pre-arranged timing of clinical encounters as it provided them with a sense of security and created an opportunity for participatory guidance [41]. Previous studies demonstrate that continuity-of-care increases client satisfaction and has been shown to decrease health service use [42–44]. We recommend structuring models of care to promote consistency of caregivers and pre-scheduled wellness check-ups completed in the home. The frequency and intensity of follow-up should be determined in parallel with HC clients to avoid unnecessary burdens, given that one participant was overwhelmed with the number of clinical encounters. With this participant, multiple professional services in addition to daily personal support were involved, resulting in more than one visit on some days. HC visits have previously been reported as intrusive and disruptive to one’s normal routine, and described as an invasion of space and time [45]. Shared-decision making is preferred by clients and may have mitigated this burden pre-emptively [46].

## Limitations

This was a multi-site qualitative study conducted across three provinces. There were several challenges to recruitment and contacting eligible participants. We attempted contact five times spread out over several days, though we were unable to contact a number of clients. Our sample included more participants from the province of Ontario, and as such, the findings presented here may depict more of an Ontario context. Control clients were not included in the design of this qualitative study; therefore, we cannot compare or comment on self-management experiences in absence of the intervention. We elected to include the perspectives from a caregiver if they participated in the intervention and were performing self-management activities. We recognize that the caregiver perspective may not represent that of the person, however the caregiver perspective in these unique instances do contribute to understanding self-management care.

## Conclusions

This study provides insight on a comprehensive cardio-respiratory self-management model from the perspective of HC clients. Overall, participants reported positive experiences with components of the DIVERT-CARE intervention. The identified strengths of our model of care included prolonged health education, comprehensive care in the home, the use of client decision aids, and continuity-of-care with dedicated providers. Our findings support that self-management programs should be offered over a sufficient period to establish trust, should provide opportunities to reinforce health learnings which can mitigate trial and error behaviours. Further, as conditions vary over time, there is a need for re-education and tailoring to changing conditions. The HC sector has the potential to provide effective strategies for health promotion by leveraging existing tools in the provision of proactive care to the client population. These factors should be taken into consideration when developing future models of care in the community.

## Supplementary Information

**Additional file 1. **This document shows the semi-structure interview guide used with clients or caregivers in the study.

**Additional file 2. **This document shows how the COREQ checklist was fulfilled in the study.

## Data Availability

The datasets used and/or analysed during the current study are available from the corresponding author on reasonable request.

## Abbreviations

CHF: Congestive heart failure
COPD: Chronic obstructive pulmonary disease
COREQ: Consolidated Criteria for Reporting Qualitative Research
ED: Emergency departments
HC: Home care
