# Supplementary material for: A qualitative study of home care client and caregiver experiences with a complex cardio-respiratory management model
Source: BMC Geriatr. 2021 May 7;21:295. doi: 10.1186/s12877-021-02251-5 (PMC8103627; doi:10.1186/s12877-021-02251-5)
Supplement: Supplementary file 1 — Additional file 1. This document shows the semi-structure interview guide used with clients or caregivers in the study. [file 12877_2021_2251_MOESM1_ESM.docx]

Additional File 1

Interview Guide

| **Introduction Questions**  1) Could you tell me a little bit about yourself and a little about the chronic disease you are living with?  2) What did you know about this disease when you were first diagnosed?  3) Could you describe a time when you had an active role in your health care for your chronic disease?  The following questions were asked for each of the DIVERT-CARE components: Nursing, Hotline, Medication Review, Advance Care Planning, Vaccines, Plan of Care  4) Regarding the services received in the home to help manage your disease and give you some tips: Could you tell me a bit about that and how it worked for you including the good and the bad?  5) How did receiving this service change how you manage your health?  6) We would like to know more about the information you received, can you tell me about how useful/not useful you found this information?  7) Do you feel prepared to manage your symptoms?  8) Is there anything you can think of that you would change in terms of how the service went?  9) How has receiving these services and support changed how you communicated with healthcare professionals?  **Closing**  10) Given your experience over the last few months, is there anything else you would like to share about your experience that may help others who are in a similar situation as you who may not have had a chance to receive the care that you did? What suggestions might you have for others?  11) Is there anything else you can think of that you need or that would help you better manage your disease so you can stay as healthy as possible at home? |
| --- |
